# Impaired synaptic plasticity and decreased glutamatergic neuron excitability induced by SIRT1/BDNF downregulation in the hippocampal CA1 region are involved in postoperative cognitive dysfunction

**DOI:** 10.1186/s11658-024-00595-5

**Published:** 2024-05-23

**Authors:** Wei-Feng Wu, Chen Chen, Jia-Tao Lin, Xin-Hao Jiao, Wei Dong, Jie Wan, Qiang Liu, Yong-Kang Qiu, Ao Sun, Yi-Qi Liu, Chun-Hui Jin, He Huang, Hui Zheng, Cheng-Hua Zhou, Yu-Qing Wu

**Affiliations:** 1 Jiangsu Province Key Laboratory of Anesthesiology, NMPA Key Laboratory for Research and Evaluation of Narcotic and Psychotropic Drugs, Xuzhou Medical University, Xuzhou, 221004 China; 2 Department of Anesthesiology, National Cancer Center/National Clinical Research Center for Cancer/Cancer Hospital, Chinese Academy of Medical Sciences and Peking Union Medical College, Beijing, 100021 China; 3 Jiangsu Key Laboratory of New Drug Research and Clinical Pharmacy, Xuzhou Medical University, Xuzhou, 221004 China

**Keywords:** SIRT1, Postoperative cognitive dysfunction, General anesthesia, Synaptic plasticity, Hippocampus

## Abstract

**Background:**

Postoperative cognitive dysfunction (POCD) is a common complication after anesthesia/surgery, especially among elderly patients, and poses a significant threat to their postoperative quality of life and overall well-being. While it is widely accepted that elderly patients may experience POCD following anesthesia/surgery, the exact mechanism behind this phenomenon remains unclear. Several studies have indicated that the interaction between silent mating type information regulation 2 homologue 1 (SIRT1) and brain-derived neurotrophic factor (BDNF) is crucial in controlling cognitive function and is strongly linked to neurodegenerative disorders. Hence, this research aims to explore how SIRT1/BDNF impacts cognitive decline caused by anesthesia/surgery in aged mice.

**Methods:**

Open field test (OFT) was used to determine whether anesthesia/surgery affected the motor ability of mice, while the postoperative cognitive function of 18 months old mice was evaluated with Novel object recognition test (NORT), Object location test (OLT) and Fear condition test (FC). The expressions of SIRT1 and other molecules were analyzed by western blot and immunofluorescence staining. The hippocampal synaptic plasticity was detected by Golgi staining and Long-term potentiation (LTP). The effects of SIRT1 and BDNF overexpression as well as chemogenetic activation of glutamatergic neurons in hippocampal CA1 region of 18 months old vesicular glutamate transporter 1 (VGLUT1) mice on POCD were further investigated.

**Results:**

The research results revealed that older mice exhibited cognitive impairment following intramedullary fixation of tibial fracture. Additionally, a notable decrease in the expression of SIRT1/BDNF and neuronal excitability in hippocampal CA1 glutamatergic neurons was observed. By increasing levels of SIRT1/BDNF or enhancing glutamatergic neuron excitability in the CA1 region, it was possible to effectively mitigate synaptic plasticity impairment and ameliorate postoperative cognitive dysfunction.

**Conclusions:**

The decline in SIRT1/BDNF levels leading to changes in synaptic plasticity and neuronal excitability in older mice could be a significant factor contributing to cognitive impairment after anesthesia/surgery.

**Graphical Abstract:**

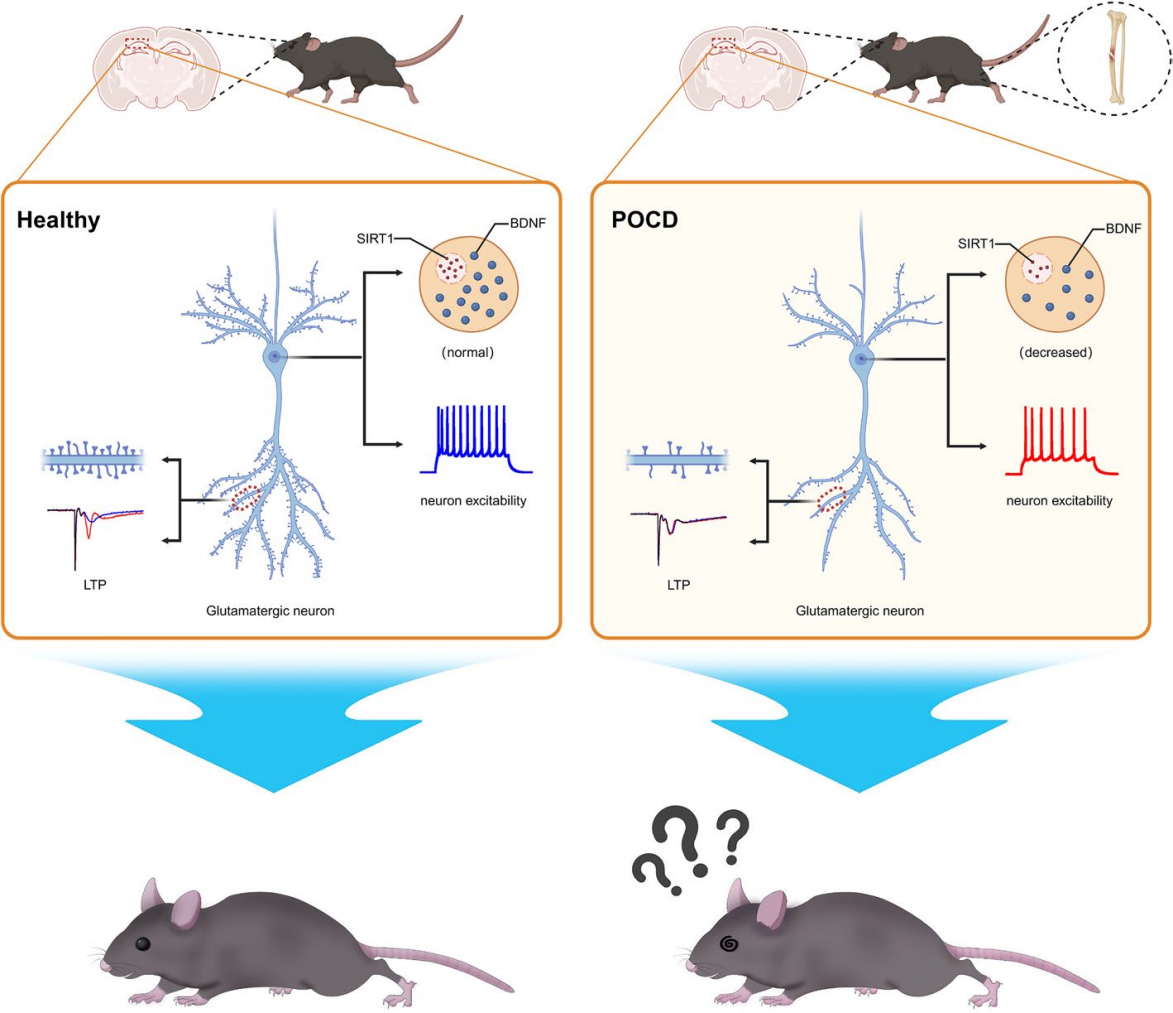

## Introduction

Postoperative cognitive dysfunction (POCD) is a frequent neurological issue that occurs in older patients following surgery. It mainly manifests as impaired cognitive functions, including the changes of learning and memory, executive function, attention, language expression, social ability and personality [1]. These issues can persist for months, and in severe cases, they may even become permanent. However, thus far, the mechanism underlying POCD is not completely clear. Previous studies have shown that the main factors affecting POCD include preoperative co-morbidities, decreased cerebral blood perfusion, anesthesia and inflammation [2–5]. Additionally, recent research has highlighted the role of synaptic plasticity in cognitive impairment [6].

Synaptic plasticity, which refers to the creation and elimination of synapses and the ability to change the electrophysiological, molecular, and structural features of synapses, is widely considered to be a key factor in regulating cognitive function [7]. The mice with impaired synaptic plasticity caused by lack expression of N-methyl-d-aspartate receptors (NMDARs) in hippocampal CA1 region display a significant deficit in spatial memory [8]. Long-term potentiation (LTP) refers to the enhancement of synapses after high-frequency electrical stimulation [9]. Induction of LTP is thought to be associated with neurons involved in learning and memory [10, 11]. Activating excitatory neuronal synaptic receptor N-methyl-d-aspartate receptors (NMDARs) is necessary for inducing LTP. Blocking hippocampal LTP with NMDARs antagonist AP5 will lead to the decrease of learning and memory ability [12]. In addition, it has been found that potassium channel subunit Kv beta 1.1 knockout mice can increase neuronal excitability and promote LTP induction, thereby improving learning and memory in elderly mice [13].

SIRT1, a type III histone deacetylase, relies on nicotinamide adenine dinucleotide (NAD^+^). SIRT1 plays a role in various physiological processes such as metabolism, inflammation, neuroprotection, and oxidative stress, through both histone and nonhistone deacetylation [14–18]. Resveratrol, an activator of SIRT1, can activate SIRT1 in aged POCD mice, reverse the decrease of sex-determining region Y-box-2 (Sox2) induced by anesthesia/surgery and alleviating cognitive decline in aged POCD mice [19].

BDNF is essential for the maintenance, support, and regeneration of neurons. It is involved in the process of LTP and promotes memory synthesis and consolidation [20]. Previous studies demonstrated that following anesthesia/surgery, mice’s hippocampal BDNF expression significantly decreased [21, 22]. Recent studies have observed that activating SIRT1 can enhance the expression of BDNF in the hippocampus and promote the formation of learning and memory [23]. It is suggested that the effect of BDNF on learning and memory is regulated by SIRT1.

Based on the above, we propose the hypothesis that anesthesia/surgery induces a decrease in hippocampal SIRT1/BDNF expression accompanied by impaired synaptic plasticity and a decrease in neuronal excitability, ultimately contributes to postoperative cognitive dysfunction.

## Materials and methods

### Animals

C57BL/6 J mice (18 months old, male, 28–32 g) were obtained from Xuzhou Medical University Animal Center for the purpose of investigating the effect of anesthesia/surgery on postoperative cognitive dysfunction. Male 18 months old VGLUT1-ires-Cre (VGLUT1) mice (Jackson Laboratory) were used to investigate the effects of SIRT1/BDNF overexpression in CA1 glutamatergic neurons and chemogenetic activation of glutamatergic neurons on POCD. Every mouse was kept on a 12-h light/dark schedule. The temperature and humidity were maintained at 22–25 ℃ and 40–60%. Food and water were freely available.

### POCD mouse model

As in previous studies [24, 25], the POCD model was established by the intramedullary fixation of tibial fracture induced by isoflurane anesthesia. Anesthesia was initiated using 3.0% isoflurane and maintained with 1.5% isoflurane throughout the procedure. To reveal the bone, a minor cut was created in the skin of the mouse’s left hindlimb. The needle for intramedullary fixation was placed through the tibia tuberosity into the medullary canal. Performing osteotomy at the mid and distal regions of the tibia, carefully suturing the wound after the operation. A heating pad was utilized during the procedure to regulate the mice’s body temperature within the range of 36 to 37 °C. After the operation, the mice were placed in a suitable environment to recover. When the mice were in stable condition, 2% lidocaine solution was locally used to treat postoperative pain, and then put into the original cage.

### Viral microinjection

The viral vectors rAAV–CaMKIIα-EGFP-WPRE, rAAV-CMV-DIO-mCherry-WPRE-hGH, AAV-CMV-DIO-SIRT1-P2A-mCherry-WPREs, rAAV-CMV-DIO-EGFP, and rAAV-CMV-DIO-BDNF-EGFP were sourced from BrainVTA Company in Wuhan, China, while pAAV-EF1α-DIO-mCherry, pAAV-EF1α-DIO-hM3D(Gq)-mCherry-WPRE were acquired from OBiO Technology Company in Shanghai, China. The virus was injected into the CA1 region (AP: − 1.95 mm; ML: ± 1.4 mm; DV: − 1.4 mm) using bilateral stereotaxic injection, with an infusion rate of 0.05 μL/min. The success of virus transfection was verified by the fluorescence signal.

### Open field test (OFT)

OFT was utilized to test whether the mice’s motor ability was impacted by the anesthesia/surgery. The mice were placed in a (50 × 50 × 50 cm^3^) square site to adapt to the environment for 10 min, and OFT was carried out 1 h later, allowing them to explore 5 min freely in the square open field. ANY-maze (ANY-maze, Stoelting Co., IL, USA) was used to record the movement track of mice. After each mouse was tested, 75% ethanol was used to wipe the open field to prevent odor from affecting the mice.

### Object location test (OLT) and novel object recognition test (NORT)

Short-term spatial and nonspatial memory in mice were assessed using the OLT and NORT according to the previous research [26, 27]. Two cylinders (A1 and A2) were selected and placed in the open field, each cylinder of the same material, size and color. The mice were placed in the open field and allowed to freely investigate the two objects for a duration of 10 min. Then, the mice were put into their own cages, and the OLT test began 1 h later. The mice were put back into the open field, but the A2 in the two cylinders had been placed in a new space. The mice were then permitted to freely explore the open field for a period of 5 min before being returned to their original cages. The NORT test was performed 24 h after the OLT test. The NORT test is similar to the OLT, except that in the test phase, the A2 in the two cylinders is replaced by a new object, A3, without changing its position. The mice are also given five minutes to freely investigate the object in the open field. Following each mouse’s behavioral test, the open field was cleaned with 75% alcohol, thereby mitigating the potential impact of residual odors from the mice on the experimental outcomes.

ANY-maze software recorded the mice’s actions, defining exploratory behavior as when the mice sniffed or touched the object within a 2 cm distance. In each experiment, an experimenter used a stopwatch to record how long the mice explored the object. Recognition memory is evaluated by the discrimination index (DI). The formula is as follows: exploration time for new locations or novel objects (%) = [exploration time for new locations or novel objects/(exploration time for new locations or novel objects + exploration time for original locations or familiar objects)] × 100% [27–29].

### Fear conditioning test (FC)

The fear conditioning test adopted a previous experimental protocol [24]. The whole process is divided into two stages: the training stage of establishing long-term memory and the testing stage [30]. The mice underwent FC training on the second day after anesthesia/surgery. Each mouse was given 6 pairs of conditioned stimuli (20-s, 70-dB tone conditioned stimulation, 25-s contextual interval) and unconditional stimuli (2-s, 0.70-mA electrical footshock) after 2 min of indoor adaptation. Random intervals of 45–60 s separated every set of conditioned and unconditional stimuli. The FC context test was performed 24 h after the FC training phase. The tone test was carried out 1 h after the context test. The context test assessed memory dependent on the hippocampus, while the tone test assessed memory independent of the hippocampus. The mice were once more placed in the training phase’s conditioned response chamber during the context test phase, but this time they were not given any stimulation. Permitting each mouse to freely explore for a duration of 5 min. The mice moved to a new conditioned response room two hours after the test ended so that they were given five minutes to explore freely. Within this time, the mice were given 3 min of tone stimulation. The software from Med Associates, Inc., USA, recorded the freezing time for each mouse during the test.

### Western blotting

The hippocampal tissue was placed in the RIPA lysate with PMSF protease inhibitor for homogenization, followed by centrifugation at 12,000 rpm at 4 ℃ to collect the supernatant. A Bicinchoninic acid (BCA) Protein Assay Kit (Beyotime, P0010, Shanghai, China) was used for measuring the protein concentration, and the proteins were subsequently treated with RIPA lysis buffer for further processing. The sample was then boiled for 10 min, cooled and subjected to sodium dodecyl sulphate–polyacrylamide gel electrophoresis (SDS-PAGE) for separation. The protein that has been isolated was moved onto a polyvinylidene fluoride (PVDF) membrane from Merck Millipore (ISEQ00010, USA). After an hour of using 5% skim milk to block the PVDF membrane. And then it was exposed to primary antibodies: SIRT1 (1:1000, 9475S, Cell Signaling Technology), BDNF (1:1000, ab108319, Abcam), and GAPDH (1:1000, ET1601-4, HUABIO) overnight in cold storage at 4 ℃. The membranes were then treated with an antibody conjugate with horseradish peroxidase (1:2000, Beyotime) for an hour at room temperature. The protein bands were then subjected to a quantitative analysis utilizing ImageJ software and an Enhanced Chemiluminescence (ECL) detection system (Beyotime).

### Immunofluorescence

Mice were first perfused with a solution of 0.9% saline and then with 4% paraformaldehyde. The brain was rapidly extracted, treated with 4% paraformaldehyde for 6–8 h, and then immersed in a with 30% sucrose solution for 3 days to dehydrate. Mouse brains were cut into slices 30 μm thick which contain hippocampus using a frozen microtome (CM1950, Leica Microsystems). Following PBS washing, the brain slices were immersed in 10% goat serum at 37 ℃ for 1 h to prevent binding of non-specific epitopes [31]. Subsequently, the brain slices were immersed in primary antibody for a duration of 72 h at 4 °C. The primary antibodies utilized were anti-SIRT1 (1:100, 9475S, Cell Signaling Technology) combined with anti-Glutamate decarboxylase 67 (GAD67) (1:300, ab26116, Abcam) and anti-SIRT1 combined with anti-Calcium/calmodulin-dependent protein kinase II (CamKIIα) (1:300, 50049S, Cell Signaling Technology), anti-BDNF (1:100, ab108319, Abcam) combined with anti-GAD67 and anti-BDNF combined with anti-CamKIIα, anti-c-Fos (1:500, 2250S, Cell Signaling Technology) combined with anti-CamKIIα. The brain sections were cleaned and immersed at 37 ℃ for 1 h with fluorescent secondary antibody. Fluorescent secondary antibodies (ab150077, ab150115, ab150113, ab150080, Abcam) were utilized at a dilution of 1:400. The nucleus was stained with a 4’,6-diamidino2-phenylindole (DAPI)-containing tablet (ab104139, Abcam). The immunofluorescence results were observed by confocal microscopy (FV1000, Olympus). Six mouse brains were taken from each group, and one brain slice was taken from each mouse as a sample (*n* = 6).

### Golgi staining

Following the manufacturer’s instructions provided by the FD Fast Golgi Staining Kit (PK401A, FD NeuroTechnologies, Inc.), the mouse brains were promptly extracted during isoflurane anesthesia and submerged in the pre-configured combination of liquid A and liquid B. The blend was altered following a 24-h storage period at room temperature and subsequent soaking for 14 days at the same temperature. Following a 3-day immersion in liquid C, the mouse brains were dehydrated and then cut into 120 μm coronal sections with a vibrating microtome (VT1000S, Leica Microsystems). The brain slices were affixed to slides coated with gelatin; the slides were then soaked in a mixture of liquid D and liquid E for 10 min. Following immersion, the slides were dehydrated in ethanol solution, made transparent with xylene, and subsequently covered evenly with neutral resin on each brain slice. An Olympus BX53 microscope was used to see the dendrites and dendritic spines of neurons in the CA1 area. Three mice were chosen from each group for Golgi staining, with three brain slices selected from each individual mouse’s brain. For each brain segment, two neurons in the hippocampal CA1 region were observed and analyzed using ImageJ software, for a total of 18 neurons in each group (*n* = 18).

### Whole-cell patch clamp recordings

Three weeks after injection of rAAV-CaMKIIα-EGFP-WPRE into CA1 region, the mice were anesthetized using isoflurane. Subsequently, the brain was promptly extracted after perfusion with a cold high glucose solution containing (mM): 80 NaCl, 3.5 KCl, 4.5 MgSO_4_, 0.5 CaCl_2_, 1.25 NaH_2_PO_4_·2H_2_O, 90 sucrose, 25 NaHCO_3_, and 10 glucose. The mouse brain was cut into slices containing the hippocampus (300 μm) using a vibrating microtome (VT1200s, Leica, Germany) at a speed of 0.12 mm/s in a high-glucose solution with oxygen. Brain slices were placed in artificial cerebrospinal fluid (ACSF) to incubate for one hour. A patch electrode (4‒8 MΩ) was used for recording. The signal is collected by MultiClamp 700B amplifier, and the data are collected and analyzed by Digidata 1550B and pClamp10.7 from Molecular Devices, Sunnyvale, USA.

### Long-term potentiation (LTP) recording

As per previous research, a vibrating microtome was used to slice the mouse brain into coronal sections that were 300 μm thick. These sections were then incubated in ACSF for one hour. Electrodes were used to stimulate the Schaffer collateral branches, and the field excitatory postsynaptic potential (fEPSP) was recorded by gently inserting a glass microelectrode filled with ACSF into the CA1 area. Following a 30-min recording of the stable fEPSP (recorded as the stable baseline), three trains of theta burst stimulation (TBS) were administered, each lasting 1 s at a frequency of 100 Hz with an interval of 20 s. Then, the fEPSP was recorded for 1 h, the change in slope was observed, and the change in the average slope of last 20 min were analyzed.

### Chemogenetic manipulation

According to prior experimental studies [32, 33], chemogenetic virus was injected into the VGLUT1 mice’s CA1 region 3 weeks before anesthesia/surgery, followed by intramedullary fixation of tibial fractures. Before electrophysiological recording, immunofluorescence and behavioral tests post-anesthesia/surgery, the virus was activated by intraperitoneal injection of pre-configured Clozapine-N-oxide (CNO, Sigma-Aldrich, Saint Louis, MO, USA). CNO was prepared by dissolving in dimethyl sulfoxide (DMSO) and diluting with normal saline, with a dosage of 3 mg/kg for each mouse.

### Statistical analysis

GraphPad Prism 8.0 is used to analyze all data and generate statistical graphs. The results of the analyzed data were represented as mean ± SEM. Comparing the differences between the two groups was carried out with the unpaired *t* test. Two-way ANOVA and Bonferroni test assessed the impact of two factors on the results. It was deemed statistically significant when *p* < 0.05.

## Results

### General anesthesia/surgery induced postoperative cognitive dysfunction

**Fig. 1:**
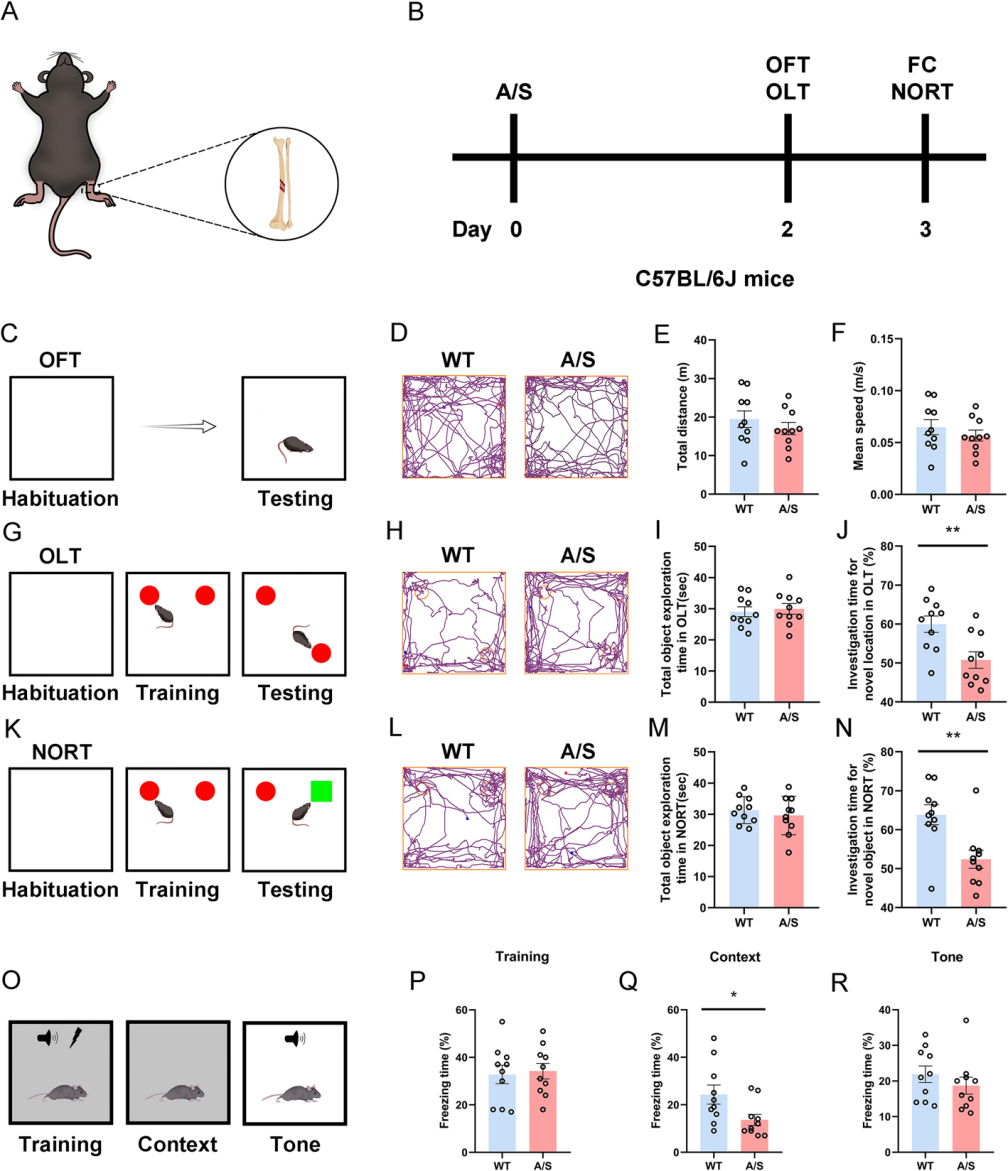
Anesthesia/surgery induced cognitive impairment in mice. Behavioral tests were performed on the second and third days after tibial fracture surgery to assess the cognitive function of the mice. **A** Diagram of intramedullary fixation of tibial fracture. **B** The behavioral timeline. **C** Experiment diagram of the OFT. **D** Representative track diagrams in the OFT. **E** Total moving distance of mice in the OFT (*n* = 10). **F** The average speed of mice in the OFT (*n* = 10). **G** Experiment diagram of the OLT. **H** Representative track diagrams in the OLT. **I** Total object exploration time in the OLT (*n* = 10). **J** The percentage of time spent exploring new location objects in the OLT **p < 0.01 (*n* = 10). **K** Experiment diagram of the NORT. **L** Representative track diagrams in the NORT. **M** Total object exploration time in the NORT (*n* = 10). **N** The percentage of time spent exploring new objects in the NORT **p < 0.01 (*n* = 10). **O** Experiment diagram of the FC. **P** Percentage of freezing time of mice during the FC training (*n* = 10). **Q** Percentage of freezing time of mice during the FC context test *p < 0.05 (*n* = 10). **R** Percentage of freezing time of mice during the FC tone test (*n* = 10)

Previous studies [24, 25] reported that mice exhibit significant postoperative cognitive impairment on the third day after tibial fracture intramedullary fixation (Fig. 1A). Therefore, we performed OFT and OLT on the second day after surgery, and then NORT and FC were performed on the third day after anesthesia/surgery (Fig. 1B).

The motor capacity of mice was assessed by OFT after anesthesia surgery (Fig. 1C, D), with the results indicating no notable disparity in total distance and average speed between WT and POCD mice, suggesting unaffected movement ability post general anesthesia/surgery (Fig. 1E, F). Subsequently, the impact of learning and memory on mice was evaluated by OLT and NORT (Fig. 1G–N). The total object exploration times for the two groups during the OLT did not differ statistically significantly (Fig. 1I). However, the control group mice were more inclined to explore objects in new locations, while the POCD group mice did not exhibit a strong preference for the two objects (Fig. 1J). Similarly, findings from the NORT indicated that there was no notable disparity in the total time spent investigating new and old objects between the two groups (Fig. 1M), but the control group took longer to explore new objects (Fig. 1N). In the fear condition test (FC) (Fig. 1O), there was no statistical difference in freezing time during the training phase (Fig. 1P). In context test, the freezing time of POCD mice was shorter than that of control mice (Fig. 1Q), which indicated that the hippocampus-dependent memory was markedly impaired in mice after anesthesia/surgery. However, there was no notable disparity in hippocampus-independent memory assessed by tone test (Fig. 1R).

### Effect of anesthesia/surgery on hippocampal SIRT1/BDNF expression

**Fig. 2:**
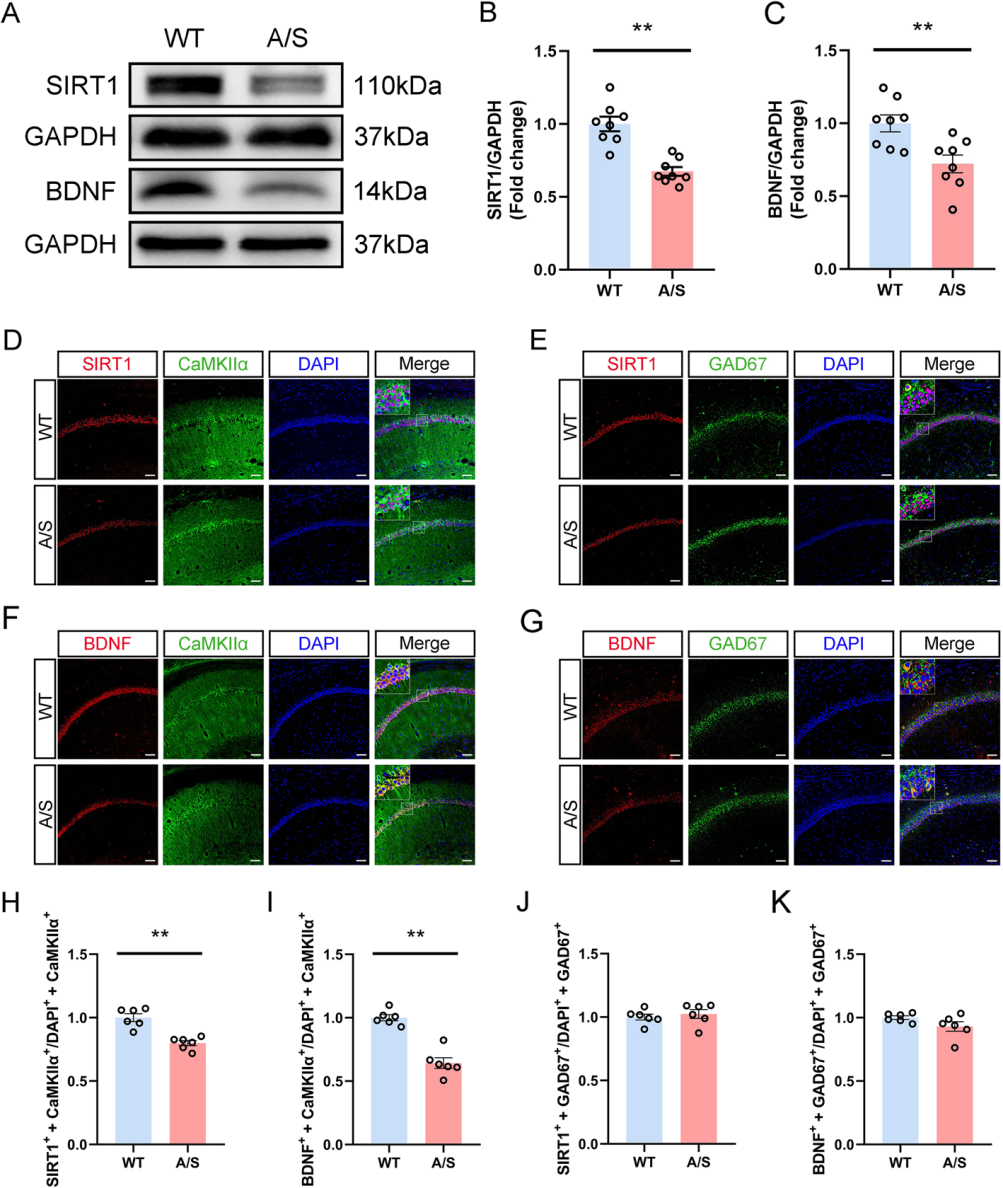
Effects of anesthesia/surgery on SIRT1/BDNF expression in the hippocampal CA1 region of POCD mice. **A** Representative Western blot protein bands. **B** The level of SIRT1 in the hippocampal CA1 region of POCD mice was decreased **p < 0.01 (*n* = 8). **C** The level of BDNF in the hippocampal CA1 region of POCD mice was decreased **p < 0.01 (*n* = 8). **D** Representative images of SIRT1 and CaMKIIα. **E** Representative images of SIRT1 and GAD67. **F** Representative images of BDNF and CaMKIIα. **G** Representative images of BDNF and GAD67. Scale bar, 100 μm. **H** Quantitation of the co-labeling rate of SIRT1 and CaMKIIα **p < 0.01 (*n* = 6). **I** Quantitation of the co-labeling rate of BDNF and CaMKIIα **p < 0.01 (*n* = 6). **J** Quantitation of the co-labeling rate of SIRT1 and GAD67 (*n* = 6). **K** Quantitation of the co-labeling rate of BDNF and GAD67 (*n* = 6)

Following the behavioral experiment, we assessed the levels of SIRT1/BDNF in the CA1 region of mice (Fig. 2A). The findings indicated a notable decrease in SIRT1 expression in POCD mice compared to WT mice (Fig. 2B). The expression of BDNF also decreased significantly (Fig. 2C). The levels of SIRT1/BDNF in glutamatergic neurons or GABAergic neurons were observed by immunofluorescence double labeling of SIRT1/BDNF with CaMKIIα or SIRT1/BDNF with GAD67 respectively (Fig. 2D–G). Fluorescence results showed that co-labeling rates of SIRT1/BDNF in glutamatergic neurons decreased significantly in mice post-anesthesia/surgery (Fig. 2H, I). However, there was no statistical difference in co-labeling rates in GABAergic neurons between the two groups (Fig. 2J, K). The findings indicate that SIRT1/BDNF expression within the CA1 region’s glutamatergic neurons was downregulated following anesthesia/surgery.

### Synaptic plasticity impairment in the hippocampal CA1 region induced by anesthesia/surgery

**Fig. 3:**
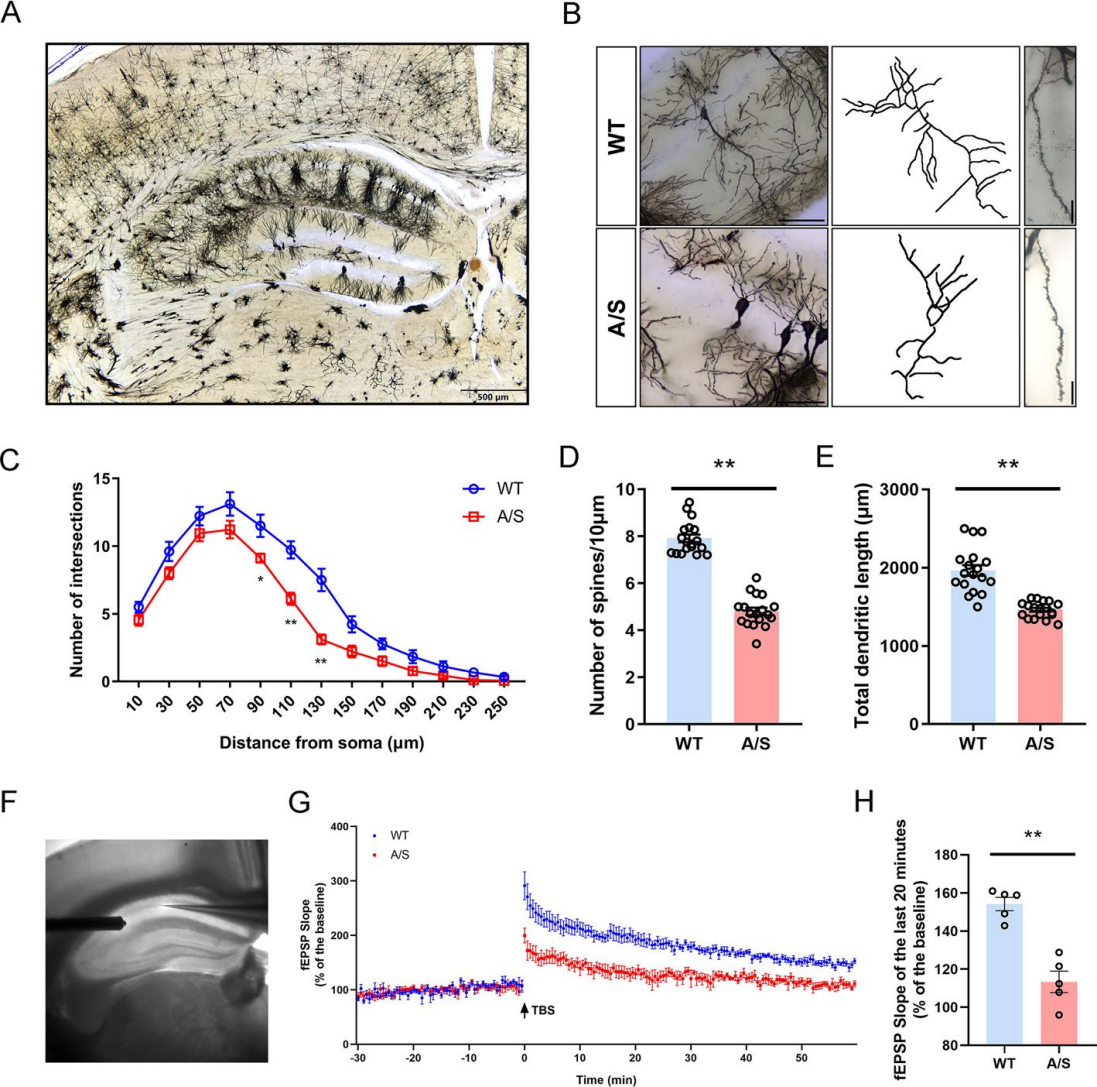
Impairment of synaptic plasticity in the hippocampal CA1 region of POCD mice. **A** Golgi staining images of 4 × in the hippocampus under electron microscope. Scale bar, 500 μm. **B** Profile images of Golgi staining of neurons in the hippocampal CA1 region. 20 × with camera tracings and 60 × for spine counting. Scale bar, 100 μm for 20 ×; 10 μm for 60 ×. **C** Quantification of dendritic intersections *p < 0.05, **p < 0.01 (*n* = 18). **D** Quantitation of the dendritic spine density **p < 0.01 (*n* = 18). **E** Quantitation of the total dendritic length **p < 0.01 (*n* = 18). **F** The sample image shows stimulation given at the location of the lateral Schaffer branch and recorded in the CA1 region. **G** LTP recorded in the CA1 region of the hippocampus. The arrow indicates the point in time of the TBS. **H** Average field excitatory postsynaptic potential (fEPSP) slope during the last 20 min after TBS. **p < 0.01 (*n* = 5)

POCD is significantly influenced by synaptic plasticity [22, 25, 34]. To investigate the impact of anesthesia/surgery on synaptic plasticity, we used Golgi staining to analyze changes in the number of dendritic branching, total dendritic length, and dendritic spine density of neurons in the CA1 region (Fig. 3A, B). The Sholl analysis results indicate that, compared to the control group mice, anesthesia/surgery decreased the dendritic branching number in the CA1 region neurons at a distance of 90–130 μm from the cell body (Fig. 3C). In addition, the dendritic spine density (Fig. 3D) and the total length of dendrites (Fig. 3E) were significantly reduced. The synaptic functional plasticity in CA1 was detected by LTP. The result showed that the hippocampal fEPSP slope after TBS was significantly decreased in mice of A/S group compared to that in control group (Fig. 3G, H). It was indicated that synaptic plasticity of neurons in the mice’s CA1 region is significantly impaired after anesthesia/surgery.

### The excitability of glutamatergic neurons was decreased in hippocampus after anesthesia/surgery

**Fig. 4:**
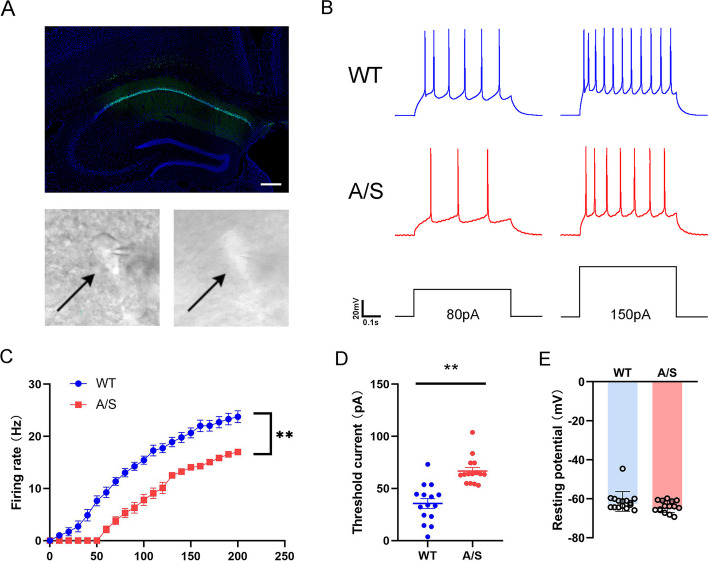
Decreased excitability of glutamatergic neurons in the hippocampal CA1 region of POCD mice. **A** Fluorescence images show the effective expression of the AAV-CaMKIIα vector in the hippocampal CA1 region and the schematic diagram of glutamatergic neurons in the hippocampal CA1 region recorded under microscope in acute brain sections. **B** Schematic diagram of action potential induced by depolarization current. **C** Fring rate of action potentials evoked by depolarizing current pulses of 0–200 pA **p < 0.01 (*n* = 15). **D** Threshold current used to excite the first action potential **p < 0.01 (*n* = 15). **E** Comparison of resting membrane potential between the two groups of mice (*n* = 15)

The mice’s CA1 area was injected with an AAV virus containing CaMKIIα to label glutamatergic neurons (Fig. 4A). The action potential of fluorescently labeled glutamatergic neurons was recorded by whole-cell patch clamp. The anesthesia/surgery group displayed a significant decrease in action potential frequency (Fig. 4B, C), and the threshold current increased (Fig. 4D), but there was no notable change in resting membrane potential (Fig. 4E). It was suggested that anesthesia/surgery result in a reduction of excitability in CA1 region glutamatergic neurons in mice.

### Effects of specifically increasing SIRT1 in CA1 glutamatergic neurons on hippocampal synaptic plasticity and neuronal excitability in POCD mice

**Fig. 5:**
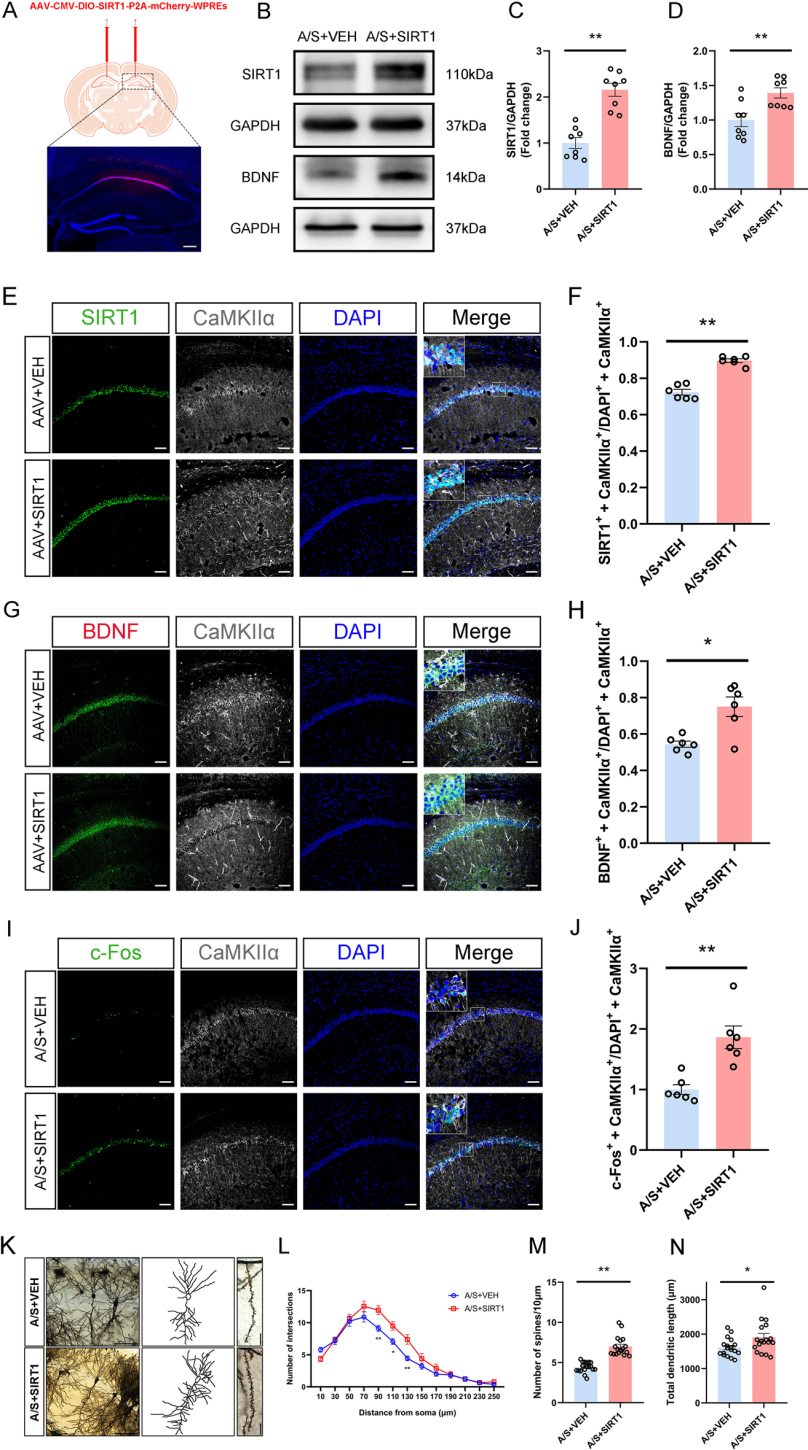
Effects of SIRT1 overexpression in hippocampal CA1 glutamatergic neurons on SIRT1/BDNF expression, synaptic plasticity and neuronal excitability. **A** Fluorescence images showed that the AAV-SIRT1 vector was effectively expressed in the CA1 region of the hippocampus. **B** Representative Western blot protein bands. **C** Quantitative results show that the level of SIRT1 in the hippocampal CA1 region of SIRT1-overexpression mice was increased **p < 0.01 (*n* = 8). SIRT1 expression in each sample was normalized to that of GAPDH. The SIRT1 level of control mice was set to 1 for quantification. **D** Quantitative results show that the level of BDNF in the hippocampal CA1 region of SIRT1-overexpression mice was increased **p < 0.01 (*n* = 8). BDNF expression in each sample was normalized to that of GAPDH. The BDNF level of control mice was set to 1 for quantification. **E** Representative images of SIRT1 and CaMKIIα. Scale bar, 100 μm. **F** Quantitation of the co-labeling rate of SIRT1 and CaMKIIα **p < 0.01 (*n* = 6). **G** Representative images of BDNF and CaMKIIα. Scale bar, 100 μm. **H** Quantitation of the co-labeling rate of BDNF and CaMKIIα *p < 0.05 (*n* = 6). **I** Representative images of c-Fos and CaMKIIα. Scale bar, 100 μm. **J** Quantitation of the co-labeling rate of c-Fos and CaMKIIα **p < 0.01 (*n* = 6). **K** Profile images of Golgi staining of neurons in the hippocampal CA1 region. 20 × with camera tracings and 60 × for spine counting. Scale bar, 100 μm for 20 ×; 10 μm for 60 ×. **L** Quantification of dendritic bifurcations *p < 0.05, **p < 0.01 (*n* = 18). **M** Quantitation of the dendritic spine density **p < 0.01 (*n* = 18). **N** Quantitation of the total dendritic length *p < 0.05 (*n* = 18)

The CA1 area of VGLUT1 mice was injected with an AAV virus carrying the SIRT1 overexpression sequence (Fig. 5A). After 21 days of virus expression, both groups of mice underwent anesthesia surgery at the same time. Figure 5B shows the representative bands of Western blot. The findings demonstrated that the hippocampus of POCD mice with overexpressed SIRT1 had a much higher expression level of SIRT1/BDNF (Fig. 5C, D). Immunofluorescence results also suggested that the co-labeling rates of SIRT1/BDNF in hippocampal CA1 glutamatergic neurons increased significantly (Fig. 5E–H). In addition, the co-labeling rates of c-Fos in hippocampal CA1 glutamatergic neurons also increased significantly (Fig. 5I, J). The results of Golgi staining (Fig. 5K) showed that the number of dendritic bifurcations in hippocampal CA1 region neurons increased significantly at 90–130 μm from the cell body after overexpression of SIRT1 (Fig. 5L), and the dendritic spine density (Fig. 5M) and the total length of dendrites (Fig. 5N) also recovered significantly. The above results show that overexpression of SIRT1 in the CA1 region can increase the expression of SIRT1 and BDNF, improve synaptic plasticity, and restore neuronal excitability.

### Effects of specifically increasing SIRT1 in CA1 glutamatergic neurons on cognitive function in POCD mice

**Fig. 6:**
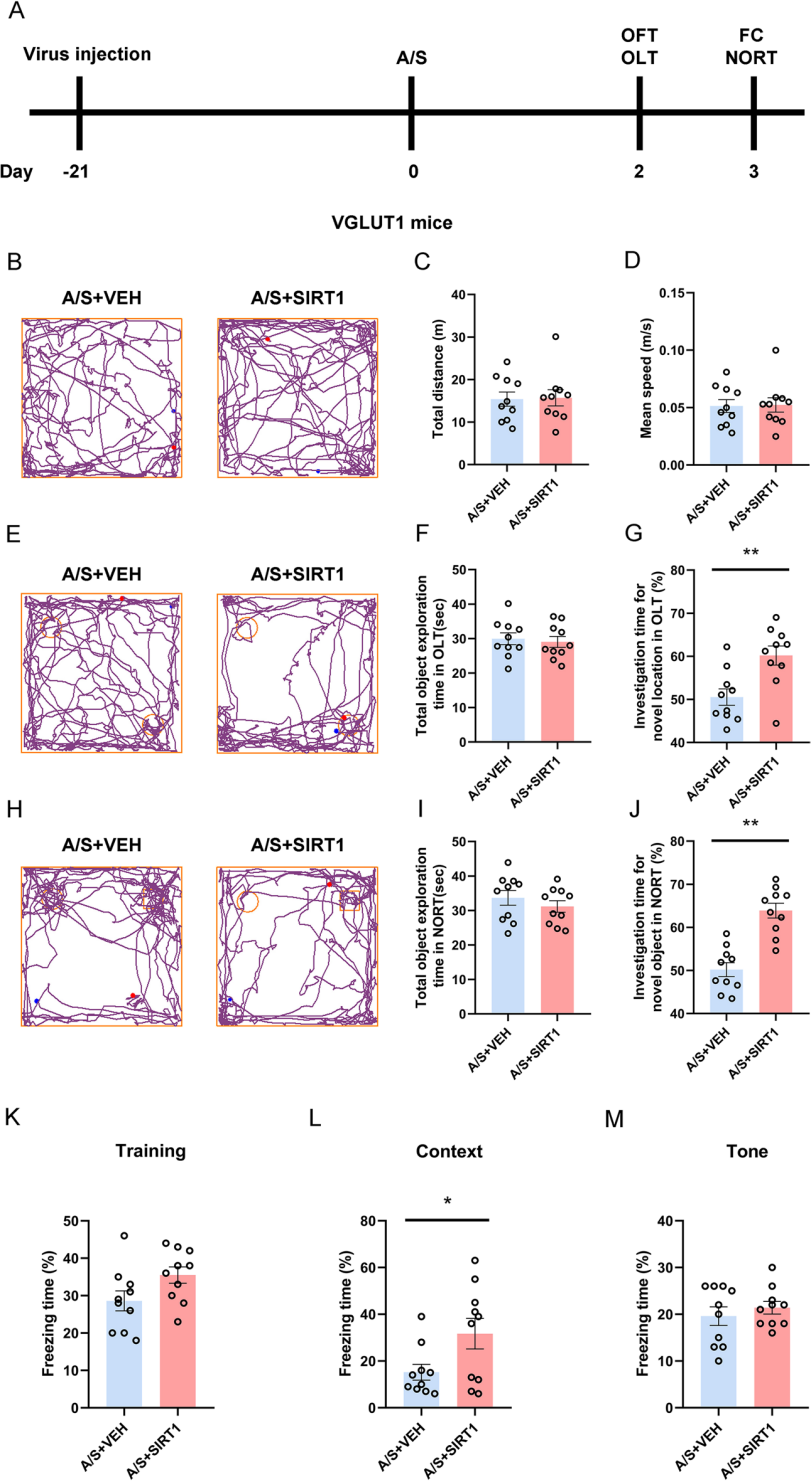
Overexpression of SIRT1 in glutamatergic neurons in the hippocampal CA1 region can improve postoperative cognitive dysfunction. **A** The behavioral timeline. **B** Representative track diagrams in the OFT. **C** Total moving distance of mice in the OFT (*n* = 10). **D** The average speed of mice in the OFT (*n* = 10). **E** Representative track diagrams in the OLT. **F** Total object exploration time in the OLT (*n* = 10). **G** The percentage of time spent exploring new location objects in the OLT **p < 0.01 (*n* = 10). **H** Representative track diagrams in the NORT. **I** Total object exploration time in the NORT (*n* = 10). **J** The percentage of time spent exploring new objects in the NORT **p < 0.01 (*n* = 10). **K** Percentage of freezing time of mice during the FC training (*n* = 10). **L** Percentage of freezing time of mice during the FC context test *p < 0.05 (*n* = 10). **M** Percentage of freezing time of mice during the FC tone test (*n* = 10)

In order to investigate the role of SIRT1 in POCD, we injected SIRT1-overexpressing virus into the hippocampal CA1 region of VGLUT1-Cre mice 3 weeks before anesthesia/surgery. Behavioral tests were conducted to assess whether specifically increasing SIRT1 expression in CA1 glutamatergic neurons could improve cognitive dysfunction (Fig. 6A). Figure 6B, E, H shows the trajectories of mice in OFT, OLT, NORT. In the OFT, compared with the control group that also underwent intramedullary fixation for tibia fracture, there was no notable disparity in the total distance (Fig. 6C) and average speed of movement (Fig. 6D). In the OLT and NORT experiments, there was no notable disparity in the total time of exploring new and old location objects (Fig. 6F) and new and old objects (Fig. 6I) between the two groups. The SIRT1-overexpression mice spent more time exploring new locations and new objects than mice in the control group (Fig. 6G, J). The FC data suggested that the two groups of mice did not show significant differences in the training and the tone test phase (Fig. 6K, M). The SIRT1 overexpression mice in context test have longer freezing time (Fig. 6L). The above behavioral results suggest that overexpression of SIRT1 significantly improves anesthesia/surgery-induced hippocampus-dependent cognitive dysfunction.

### Effects of specifically increasing BDNF in CA1 glutamatergic neurons on hippocampal synaptic plasticity and neuronal excitability in POCD mice

**Fig. 7:**
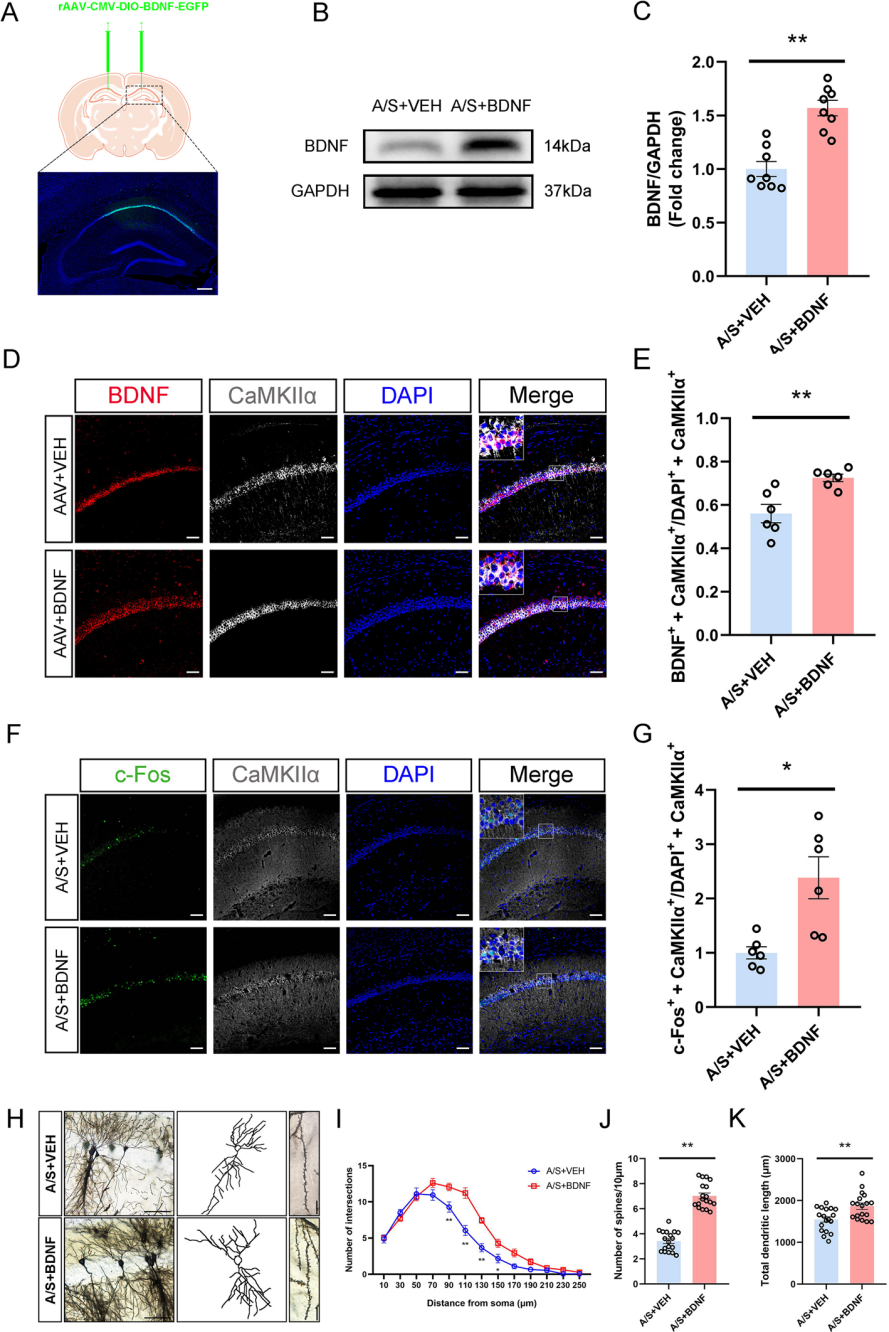
Effects of BDNF overexpression in hippocampal CA1 glutamergic neurons on BDNF expression, synaptic plasticity and neuronal excitability. **A** Fluorescence images showed that the AAV-BDNF vector was effectively expressed in the CA1 region of the hippocampus. **B** Representative Western blot protein bands. **C** Quantitative results show that the level of BDNF in hippocampal CA1 region of BDNF-overexpression mice was increased **p < 0.01 (*n* = 8). BDNF expression in each sample was normalized to that of GAPDH. The BDNF level of control mice was set to 1 for quantification. **D** Representative images of BDNF and CaMKIIα. Scale bar, 100 μm. **E** Quantitation of the co-labeling rate of BDNF and CaMKIIα **p < 0.01 (*n* = 6). **F** Representative images of c-Fos and CaMKIIα. Scale bar, 100 μm. **G** Quantitation of the co-labeling rate of c-Fos and CaMKIIα *p < 0.05 (*n* = 6). **H** Profile images of Golgi staining of neurons in the hippocampal CA1 region. 20 × with camera tracings and 60 × for spine counting. Scale bar, 100 μm for 20 ×; 10 μm for 60 ×. **I** Quantification of dendritic bifurcations *p < 0.05, **p < 0.01 (*n* = 18). **J** Quantitation of the dendritic spine density **p < 0.01 (*n* = 18). **K** Quantitation of the total dendritic length **p < 0.01 (*n* = 18)

BDNF is essential for regulating synaptic plasticity [35]. In addition, the expression of BDNF has a positive correlation with the degree of POCD [36]. Therefore, we injected an AAV virus carrying an overexpressed BDNF sequence into the CA1 region of VGLUT1 mice (Fig. 7A). After 21 days of virus expression, the level of BDNF was detected to verify the effect of virus expression (Fig. 7B–E). In addition, the co-labeling rates of c-Fos in hippocampal CA1 glutamatergic neurons also increased significantly (Fig. 7F, G). The brains of mice injected with BDNF-overexpressing virus were performed with Golgi staining (Fig. 7H). The number of dendritic branches in BDNF-overexpressing mice’s CA1 region was significantly increased at 90–150 μm from the cell body (Fig. 7I). At the same time, the dendritic spine density (Fig. 7J) and the total length of dendrites increased significantly (Fig. 7K). In conclusion, increasing BDNF levels in postoperative cognitive dysfunction mice’s CA1 region effectively mitigates synaptic plasticity impairment and concurrently enhances neuronal excitability.

### Effects of specifically increasing BDNF in CA1 glutamatergic neurons on cognitive function in POCD mice

**Fig. 8:**
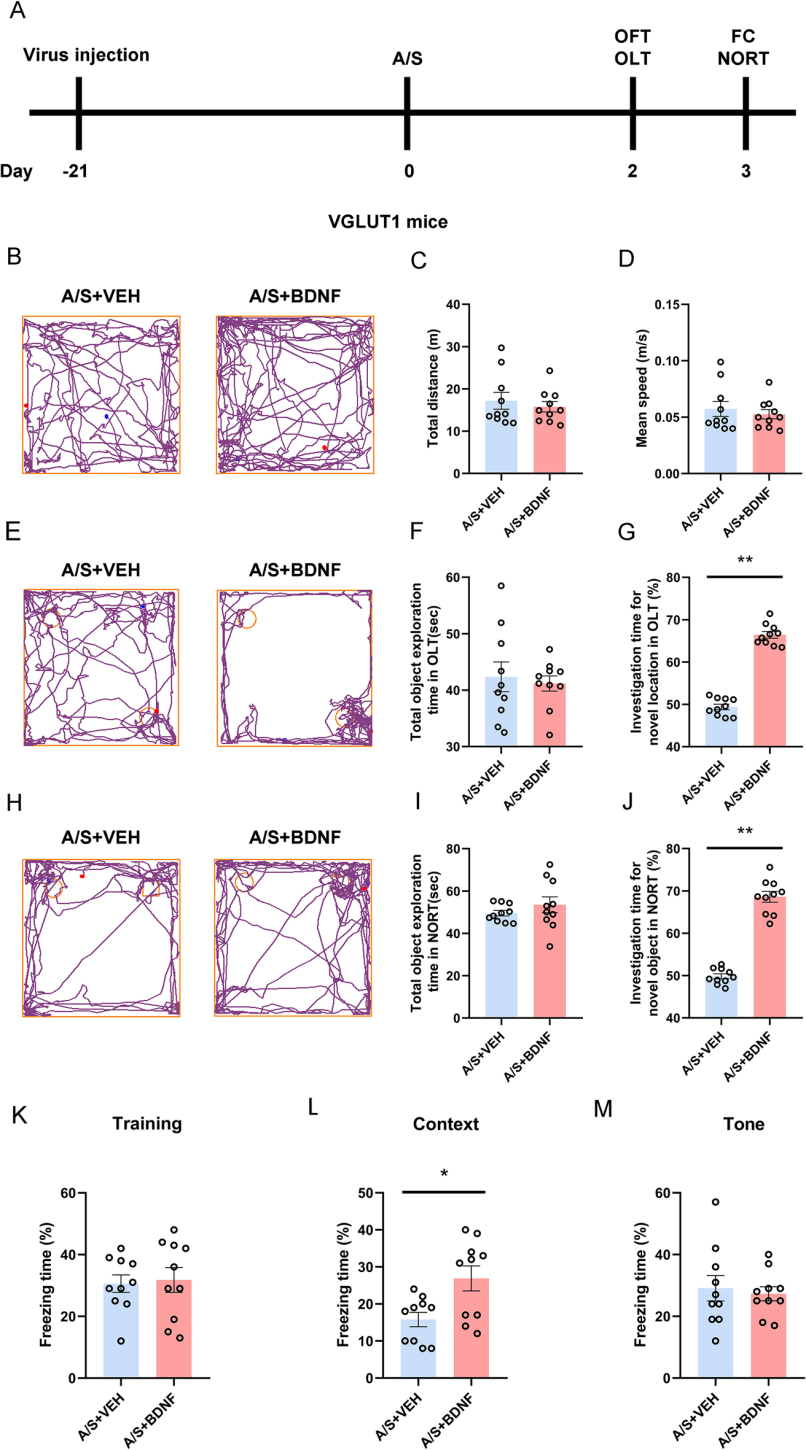
Overexpression of BDNF in glutamatergic neurons in the hippocampal CA1 region can improve postoperative cognitive dysfunction. **A** The behavioral timeline. **B** Representative track diagrams in the OFT. **C** Total moving distance of mice in the OFT (*n* = 10). **D** The average speed of mice in the OFT (*n* = 10). **E** Representative track diagrams in the OLT. **F** Total object exploration time in the OLT (*n* = 10). **G** The percentage of time spent exploring new location objects in the OLT **p < 0.01 (*n* = 10). **H** Representative track diagrams in the NORT. **I** Total object exploration time in the NORT (*n* = 10). **J** The percentage of time spent exploring new objects in the NORT **p < 0.01 (*n* = 10). **K** Percentage of freezing time of mice during the FC training (*n* = 10). **L** Percentage of freezing time of mice during the FC context test *p < 0.05 (*n* = 10). **M** Percentage of freezing time of mice during the FC tone test (*n* = 10)

VGLUT1 mice were given an injection of the BDNF overexpression virus in the CA1 area. After waiting for virus expression for 21 days, anesthesia/surgery was performed simultaneously with the control group. Behavioral adaptation and testing were performed on the second and third day after surgery (Fig. 8A). Figure 8B, E, H showed the representative track diagrams of mice in OFT, OLT, and NORT, respectively. The OFT results showed that by assessing the total distance (Fig. 8C) and average speed (Fig. 8D) of the mice, the motor ability of both groups of mice was not affected. The results of Fig. 8F, I show that both groups have the same ability to explore objects, BDNF-overexpression mice showed a longer time to explore new locations (Fig. 8G) and new objects (Fig. 8J). In the training and tone test phase of FC, there was no significant difference in the freezing time of mice from different groups (Fig. 8K, M). In the context test, BDNF-overexpression mice showed longer freezing time (Fig. 8L).

### Effect of activating hippocampal glutamatergic neurons on LTP

**Fig. 9:**
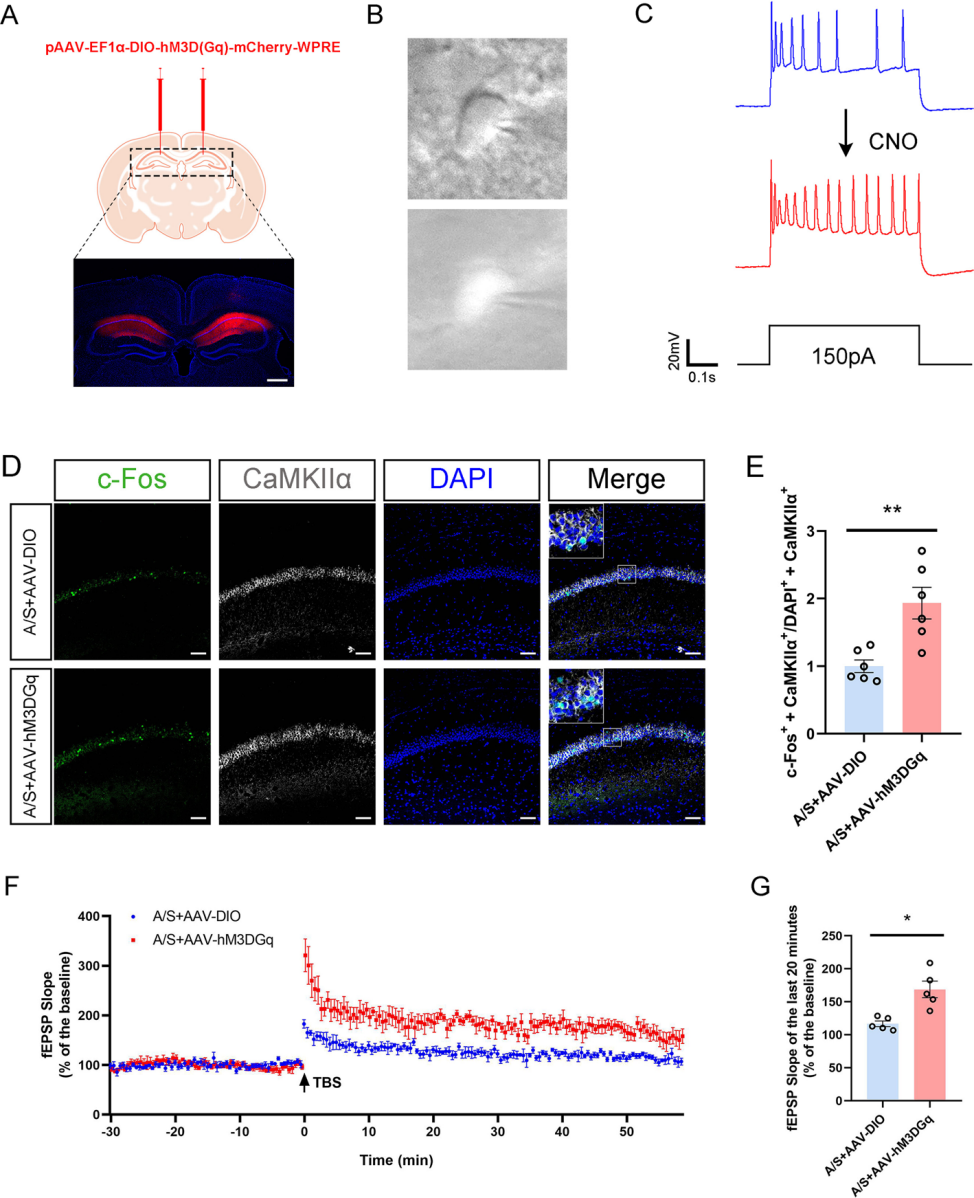
Effect of specific activation of hippocampal CA1 glutamatergic neurons on synaptic plasticity. **A** Fluorescence images showed that the chemogenetics vector was effectively expressed in the CA1 region of the hippocampus. **B** The schematic diagram of glutamatergic neurons in the hippocampal CA1 region recorded under microscope in acute brain sections. **C** In vitro infusion of CNO (10 mM) increased the firing frequency of glutamatergic neurons in the hippocampal CA1 region of AAV-hM3D(Gq)-injected POCD mice. **D** Representative colabeling images of c-Fos and CaMKIIα. Scale bar, 100 μm. **E** Quantitation of the co-labeling rate of c-Fos and CaMKIIα **p < 0.01 (*n* = 6). **F** LTP recorded in the CA1 region of the hippocampus. The arrow indicates the point in time of the TBS. **G** Average fEPSP slope during the last 20 min after TBS *p < 0.05 (*n* = 5)

In order to further explore the relationship between neuronal excitability and POCD, we injected the AAV-EF1α-DIO-hM3D(Gq)-mCherry virus into the VGLUT1 mice’s CA1 region (Fig. 9A) and anesthesia/surgery was performed 21 days after virus expression. Mice were injected intraperitoneally with CNO on the third day after anesthesia/surgery, and whole-cell patch-clamp experiments were performed to observe the depolarization and firing responses of excitatory neurons in the mice’s CA1 region (Fig. 9B). Electrophysiological results showed a significant increase in the action potential frequency of fluorescently labeled neurons, indicating that CNO effectively activated the chemogenetic virus (Fig. 9C). Following that, immunofluorescence was used to detect c-Fos expression in the mice’s CA1 region. The findings indicated a notable rise in the co-labeling rates of c-Fos in glutamatergic neurons (c-Fos^+^  + CaMKIIα^+^/DAPI^+^  + CaMKIIα^+^) was significantly increased in AAV-hM3D(Gq)-injected POCD mice, while the co-labeling rates of c-Fos in glutamatergic neurons was relatively low in control vector-injected POCD mice (Fig. 9D, E). LTP results of the hippocampal CA1 region in acute brain sections after pretreatment with CNO showed that the fEPSP slope increased after injection of chemically activated virus compared with that in the control group (Fig. 9F, G).

### Effect of activating hippocampal glutamatergic neurons on postoperative cognitive function

**Fig. 10:**
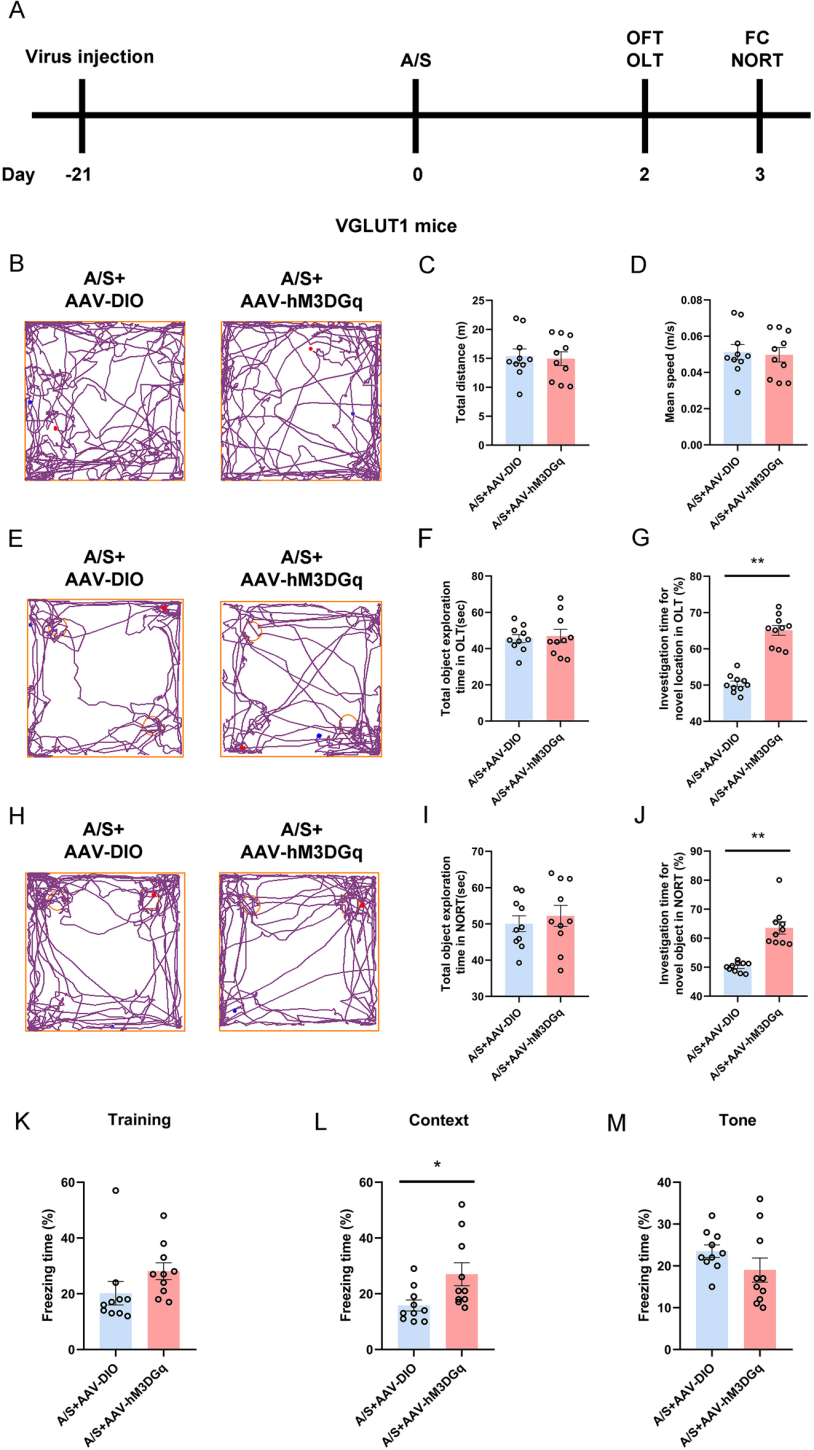
Specific activation of glutamatergic neurons in hippocampal CA1 significantly improved postoperative cognitive dysfunction in mice. **A** The behavioral timeline. **B** Representative track diagrams in the OFT. **C** Total moving distance of mice in the OFT (*n* = 10). **D** The average speed of mice in the OFT (*n* = 10). **E** Representative track diagrams in the OLT. **F** Total object exploration time in the OLT (*n* = 10). **G** The percentage of time spent exploring new location objects in the OLT **p < 0.01 (*n* = 10). **H** Representative track diagrams in the NORT. **I** Total object exploration time in the NORT (*n* = 10). **J** The percentage of time spent exploring new objects in the NORT **p < 0.01 (*n* = 10). **K** Percentage of freezing time of mice during the FC training (*n* = 10). **L** Percentage of freezing time of mice during the FC context test *p < 0.05 (*n* = 10). **M** Percentage of freezing time of mice during the FC tone test (*n* = 10)

VGLUT1 mice were given an injection of the pAAV-EF1α-DIO-hM3D(Gq)-mCherry-WPRE virus into the CA1 area. Anesthesia/surgery was performed 21 days after virus expression. Behavioral tests were performed on the second and third day after anesthesia/surgery (Fig. 10A). Figure 10B, E, H were representative track diagrams of mouse behavioral tests. In the OFT, compared with the control group that also underwent anesthesia/surgery, there was no notable disparity in the total distance (Fig. 10C) and average speed of movement (Fig. 10D). In OLT and NORT, Fig. 10F, I results showed that the two groups of mice had the same exploration ability. AAV-hM3D(Gq)-injected POCD mice showed increased time spent exploring new locations (Fig. 10G) and new objects (Fig. 10J). According to the FC data, there were no significant distinctions between the two mice groups during the training or tone test phases (Fig. 10K, M). While in the context test stage, AAV-hM3D(Gq)-injected POCD mice showed longer freezing time (Fig. 10L). These results suggest that specific activation of glutamatergic neurons can improve postoperative cognitive dysfunction.

## Discussion

Studies on cognitive function in patients after anesthesia/surgery have been conducted for more than 100 years. In 2018, "perioperative neurocognitive disorders (PND)" was recommended by the Perioperative Cognition Nomenclature Working Group as the overarching term for cognitive dysfunction in the preoperative or postoperative period. Specific components of PND include cognitive impairment diagnosed before surgery, postoperative delirium and delayed neurocognitive recovery [37–42]. As all the mice used in our experiments were healthy mice and our experiments focused on the changes in cognitive function after anesthesia/surgery, we still use the term "postoperative cognitive dysfunction (POCD)" in order to more accurately express the actual content of our study. Currently, age is recognized as the key risk factor of postoperative cognitive dysfunction (POCD), but the specific mechanism underlying POCD is still not fully determined [43, 44]. Therefore, exploring the mechanism underlying POCD is particularly important, especially in today’s aging society. This study aims to investigate whether changes in synaptic plasticity and neuronal excitability in the CA1 region, regulated by SIRT1/BDNF, are involved in POCD. This study demonstrated that reduced SIRT1/BDNF expression, impaired synaptic plasticity, and decreased neuronal excitability were observed in glutamatergic neurons in the CA1 region of mice after anesthesia/surgery, accompanied by significant cognitive impairment. Activating glutamatergic neuron excitability or overexpressing SIRT1/BDNF in glutamatergic neurons may improve this impaired synaptic plasticity and cognitive dysfunction.

Hippocampus is a key brain region for the study of spatial memory and episodic memory [45–48]. Previous studies have demonstrated that spatial memory and hippocampus-dependent memory impairment were found in mice three days after intramedullary fixation of tibial fracture, but hippocampus-independent memory was not affected [24, 25, 49]. Our experiment also proved that the freezing time of mice in the training phase and tone test phase of FC did not show significant statistical difference, but in the context test, the mice in the anesthesia/surgery group showed shorter freezing time. This shows that after anesthesia/surgery, the learning ability of mice during the training phase is not affected, but the hippocampus-dependent memory is significantly impaired.

Synaptic plasticity plays a key role in regulating cognitive function [6, 50, 51]. In the mammalian brain, long-term potentiation (LTP) of the CA1 area is a crucial model of activity-dependent synaptic plasticity [52]. Studies have found that anesthesia/surgery can induce neuroinflammation, cause synaptic plasticity impairment, and lead to POCD [25]. Appropriate exercise can improve synaptic plasticity and relieve postoperative cognitive dysfunction by relieving neuroinflammation caused by anesthesia/surgery [53]. In our study, Golgi staining and LTP experiment also proved that synaptic plasticity impairment occurred in POCD mice after anesthesia/surgery.

Glutamatergic neurons, serving as crucial afferent projection neurons in the hippocampus, play a significant role in learning and memory [54]. Recent studies have shown that activating glutamatergic neurons with blue light can effectively reduce cognitive deficits caused by repeated propofol exposure in neonatal rats [31]. Based on this theory, we investigated whether the excitatory changes of glutamatergic neurons in the mice’s CA1 region plays a role in the development of cognitive impairment after general anesthesia. Glutamatergic neurons excitability in the POCD mice’s CA1 region was assessed using a whole-cell patch clamp experiment. The findings indicated a reduction in neuronal excitability in mice with POCD. While the cognitive impairment caused by anesthesia/surgery can be alleviated by specifically activating glutamatergic neurons.

SIRT1, a homologue of SIR2, has been shown to have neuroprotective effects in central neurodegenerative diseases [55]. SIRT1 also participates in memory formation by regulating synaptic plasticity [56, 57]. It has been found that anesthesia/surgery can cause a decrease in the expression of SIRT1 [25, 58]. However, administration of the SIRT1 agonist SRT1720 in mice can reduce the level of inflammatory cells and, more importantly, was able to alleviate synaptic plasticity impairment and postoperative cognitive dysfunction [59]. In this study, we found that the expression of SIRT1 decreased after anesthesia/surgery. However, when we injected SIRT1 overexpressed virus into the VGULT1 mice’s CA1 region, the expression of SIRT1 and BDNF was significantly increased, synaptic plasticity impairment and neuronal excitability were also recovered, and the cognitive impairment induced by anesthesia/surgery was improved. It can be inferred that increasing the expression of SIRT1 can significantly improve POCD. This improvement may be attributed to increased expression of BDNF, reduction of synaptic plasticity impairment and recovery of neuronal excitability. Other studies have shown that SIRT1 has the ability to specifically bind to the lysine 464 site of Methyl-CpG binding protein 2 (MeCP2) to regulate the expression of BDNF. In mice with SIRT1 knockdown, the MeCP2 of the BDNF promoter is significantly increased while the expression of BDNF is significantly decreased [60]. These findings suggest that SIRT1 may indirectly regulates the expression of BDNF by modulating the acetylation level of MeCP2, which in turn influences synaptic plasticity and contributes to the occurrence and development of POCD. In conclusion, these results further highlight the important role of SIRT1 in POCD. Further research on SIRT1 will provide valuable insights for the prevention and treatment of this condition.

BDNF, a neurotrophic factor found throughout the brains of mammals, is crucial for the development and plasticity of glutamatergic and GABAergic synapses [61]. BDNF has been identified as a crucial molecule in promoting LTP in various brain regions, including the hippocampus [62–64]. Research has shown that BDNF is crucial in the treatment of central nervous system diseases [65–67]. Overexpression of BDNF improves cognitive function and ameliorates impairment of synaptic plasticity in mice with Alzheimer’s disease. Some studies have also discovered that neuroinflammation induced by anesthesia/surgery in mice can reduce the level of BDNF, lead to synaptic plasticity impairment and hippocampus-dependent cognitive dysfunction. Our previous experiments confirmed that after anesthesia / operation, the expression of BDNF decreased, synaptic plasticity was impaired and neuronal excitability decreased, resulting in POCD in mice. However, overexpression of BDNF in the VGLUT1 mice’s CA1 region can significantly reduce synaptic plasticity impairment, restore neuronal excitability, and alleviate postoperative cognitive decline in mice.

It is known that neuroinflammation is involved in postoperative cognitive dysfunction [5, 68]. Previous study showed that resveratrol significantly alleviated hippocampal neuroinflammation by activating SIRT1 and prevented cognitive decline after abdominal surgery in aged rats [69]. BDNF is recognized as an important regulator in neuroinflammation-induced cognitive dysfunction [70]. It was reported that neuroinflammation induced by anesthesia/surgery markedly inhibited the expression of BDNF in hippocampus, which contributed to postoperative cognitive dysfunction, while attenuating neuroinflammation by IL-1 receptor antagonists can improve postoperative cognitive function through increasing the expression of BDNF [68]. Therefore, SIRT1 may improve postoperative cognitive dysfunction by alleviating hippocampal neuroinflammation and upregulating BDNF expression.

In this study, only male mice were used to investigate the role of SIRT1/BDNF in POCD. Although many studies have suggested that sex may not be a key factor affecting POCD [71, 72], it is necessary to clarify whether the synaptic plasticity and neuronal excitability impairment mediated by SIRT1/BDNF downregulation were also the important cause of POCD in female mice. In addition, although the Golgi staining technique used in this study can directly observe the synaptic structural plasticity of neurons through microscopic images, its limitation is also obvious, since it randomly stains only a subset of neurons, providing a partial and unpredictable view of the overall neuronal population, potentially leading to incomplete or biased results. Besides Golgi staining, other techniques can also be used in the future studies to evaluate synaptic plasticity, such as immunohistochemistry for synaptophysin and synapsin, or employing in vivo imaging methods through [11C] UCB-J radiotracer, which would be valuable for a more comprehensive assessment of synaptic plasticity. Moreover, the specific mechanisms that cause changes in SIRT1/BDNF expression after anesthesia are not fully understood, so further studies on the upstream and downstream mechanisms of SIRT1 and BDNF are needed.

In conclusion, this research indicates that the reduced levels of SIRT1/BDNF and impaired synaptic plasticity in the hippocampal CA1 region after anesthesia/surgery are involved in the decrease of excitability of glutamatergic neurons and the occurrence of POCD. Overexpression of SIRT1/BDNF and specific activation of glutamatergic neurons in the hippocampal CA1 region have the potential to restore synaptic plasticity and improve postoperative cognitive impairment. The information offers fresh insights into the mechanism of postoperative cognitive dysfunction and helps pinpoint possible treatment options.

## Data Availability

The datasets used and/or analyzed during the current study are available from the corresponding author on reasonable request.

## Abbreviations

POCD: Postoperative cognitive dysfunction
SIRT1: Silent mating type information regulation 2 homologue 1
BDNF: Brain-derived neurotrophic factor
AAV: Adeno associated virus
VGLUT1: Vesicular glutamate transporter 1
LTP: Long-term potentiation
NMDARs: N-methyl-d-aspartate receptors
NAD^+^: Nicotinamide adenine dinucleotide
Sox2: Sex-determining region Y-box-2
OFT: Open field test
OLT: Object location test
NORT: Novel object recognition test
FC: Fear conditioning test
PVDF: Polyvinylidene fluoride
ECL: Enhanced Chemiluminescence
CamKIIα: Calcium/calmodulin-dependent protein kinase II
GAD67: Glutamate decarboxylase 67
DAPI: 4’,6-Diamidino2-phenylindole
ACSF: Artificial cerebrospinal fluid
fEPSP: Field excitatory postsynaptic potential
TBS: Theta burst stimulation
CNO: Clozapine-N-oxide
DMSO: Dimethyl sulfoxide
PND: Perioperative neurocognitive disorders
MeCP2: Methyl-CpG binding protein 2
AD: Alzheimer’s disease
PSD-95: Postsynaptic density 95
